# The importance of clinical and labour market histories in psychiatric disability retirement: analysis of the comprehensive Finnish national-level RETIRE data

**DOI:** 10.1007/s00127-019-01815-6

**Published:** 2019-12-05

**Authors:** S. Pirkola, J. Nevalainen, M. Laaksonen, S. Fröjd, K. Nurmela, T. Näppilä, A. Tuulio-Henriksson, R. Autio, J. Blomgren

**Affiliations:** 1 Faculty of Social Sciences, Health Sciences Unit, Tampere University, Arvo Ylpön Katu 34, 33014 Tampereen Yliopisto, Finland; 2 Department of Psychiatry, Tampere University Central Hospital, Tampere, Finland; 3 The Finnish Centre for Pensions, Helsinki, Finland; 4 Department of Psychology and Logopedics, Faculty of Medicine, University of Helsinki, Helsinki, Finland; 5 The Social Insurance Institution of Finland, Helsinki, Finland

**Keywords:** Occupational disability, Psychiatric retirement, Mental disorders, Rehabilitation, Unemployment, Labour market history, Sequence analysis, Mortality

## Abstract

**Objectives:**

Despite the stable incidence of mental disorders in Finland and Europe, mental health-related occupational disability has been increasing. We unveiled the paths to permanent psychiatric disability, recovery, or death, by analysing sequences of labour market participation.

**Methods:**

The RETIRE register database includes information regarding all persons (*n* = 42,170) awarded an ICD-10 psychiatric disability pension between 2010 and 2015 in Finland. We identified clusters of typical paths of pre-retirement labour market history. Controlling for major mental disorders, age, and sex, we evaluated factors associated with returning to work (RTW), or death, over a 5-year follow-up period.

**Results:**

Only 10.5% of the disabled subjects returned to work within the follow-up. Half of them ended up with a permanent disability pension. Seven distinguishable paths to disability were identified. Subjects in the cluster characterized by steady employment were relatively often females, lost their work ability due to affective disorders, and had the highest rate of returning to work (16.3%). Mortality was highest (9%) among the cluster characterized by long-term unemployment. Distributions of major diagnostic groups, as well as age and sex, differed between clusters. After their adjustment in the analysis of RTW or death, the identified labour market history paths prior to losing work ability remained as important independent prognostic factors for both outcomes.

**Conclusions:**

The complex retirement process involves identifiable clinical and contextual associating factors. Labour market history patterns associate with varying prognoses after psychiatric retirement. Prolonged unemployment appears as a predictor of relatively poor prognoses, whereas employment indicates the opposite.

**Electronic supplementary material:**

The online version of this article (10.1007/s00127-019-01815-6) contains supplementary material, which is available to authorized users.

## Introduction

Despite the fact that the incidence of mental disorders has been apparently stable in Finland and Europe during recent decades, the importance of occupational disability related to such conditions has been increasing in most countries [1–4]. The reasons for this involve changes in working life, large-scale cultural factors, and a failure of our services, social policy and perhaps also treatment practices, to respond to these changes [5, 6]. Research is needed for developing more effective rehabilitative services to prevent prolonged disability among the service systems of Western societies [7].

Finland is considered to be a well-developed North European welfare society, with tax-funded public services, including social and health care, and social insurance for premature occupational disability [8]. Mental disorders have been the most common cause for disability pensions since the year 2000, reaching 50% of all disability pensions in 2017 [9]. In 2017, mental disorders were also responsible for more than one-third of all new disability pensions [10]. An international evaluation by the OECD showed that the share of disability pensions granted due to mental disorders in Finland is relatively high [8]. Excess mortality is reportedly associated with disability retirement due to mental disorders, partly because of suicides and other non-natural causes [11].

The aim the current study is to characterize, among the major diagnosis-related and other sub-populations, the overall process of premature retirement due to mental disorders, and to investigate whether the employment histories are prognostic factors for two major outcomes: return to work (RTW) and death. To achieve this, we extracted a comprehensive database from the Finnish register resources, including all subjects with temporarily or permanently granted psychiatric disability pension between 2010 and 2015, complemented with population register data and data on the labour market history of the subjects. We present a sequence analysis of labour market status histories leading to disability due to mental disorders, to extract the most typical paths. This longitudinal investigation provides insight into different population groups which could benefit from diverse means of intervention to improve their recovery, in terms of higher rates of RTW or increased survival.

## Data and methods

### The disability retirement data

In the Finnish disability retirement system, individuals with problems with work ability due to medical disorders or injury may be awarded either a temporary or permanent, full or partial disability pension if certain criteria are fulfilled. The disability must be caused by a medical disorder and be severe enough to prevent the person from working. In most cases, the person first spends approximately 1 year (300 working days) on sickness allowance, with an expected possibility to return to work, unless the disability is considered severe enough for immediate retirement. Disability pensions may be paid through the earnings-related scheme or through the national pension scheme, guaranteeing a minimum level of pension for those without previous earnings.

The most common psychiatric disorders leading to disability pension are moderate-to-severe depression or psychotic disorders [12]. Examination and subsequent written statements by medical doctors (preferably psychiatrists or occupational medicine specialists) are required, and separate medical specialists and insurance physicians, serving either the National Social Insurance Institution or private insurance companies responsible for the earnings-related scheme, manage the process of granting the disability pension.

### Data sources

We obtained access to data sets from the registers of the Social Insurance Institution of Finland (SII), the Finnish Centre for Pensions (FCP), the National Institute for Health and Welfare and Statistics Finland. The data in distinct national registers can be interlinked, based on personal identification numbers (PIN) [13] which are for research purposes pseudonymized. In the present study, we utilized data from the Register of Sickness Allowances and Register on National Pensions (temporary and permanent disability pensions) of the SII, Register on Pensions (temporary and permanent disability pensions) and Employment Register (employment and unemployment periods) of FCP, and the Population Information System (gender, age) and Cause-of-Death Register (deaths) maintained by Statistics Finland.

### Study population

Our study population consisted of all persons who were granted a temporary or permanent disability pension due to a mental disorder (ICD 10: F04–F69, F80–F99) between 2010 and 2015 (*n* = 50,728). Individual disorders were categorized into larger groups, according to the ICD-10 classification system [14]. Intellectual disabilities (F70–79) were considered as non-responsive to rehabilitative intervention, and were thus excluded. Furthermore, we limited the study population by excluding all subjects (a) who did not have a mental disorder as their primary diagnosis, (b) who had received permanent disability pension due to a mental disorder prior to January 1, 2010 and (c) who had received a temporary disability pension within a year before the index admission. Thus, our final study population included 42,170 persons. For diagnostic analyses, we used the ICD-10 major diagnostic categories: F10–F19, F20–F29, F30–F39, etc., which have been commonly used in similar studies earlier [14]. The study subjects’ preceding employment and disability history up to 10 years (5 years prior to and after retirement) was included, as well as all available census data, and possible causes of death. The lengths of follow-up time-periods varied from 1 month to 10 years (median 7.6 years), being shortest for those pensioned in 2015. The database is located at Statistics Finland.

### Statistical analysis

The analysis of the data consisted of four main parts: (1) description of the study population and prevalent states before and after retirement; (2) a sequence analysis to characterize the typical paths before the retirement, and their use in, (3) an analysis of the probabilities of returning to work and (4) a survival analysis.

For the sequence analysis, we defined the time of the first psychiatric disability pension as the time origin, and 1 month as the unit of time. We analysed labour market states up to 5 years before the time origin. At each month, the ‘state’ of each individual was assigned a label: permanent psychiatric disability pension (full time or partial) [PDP(P)]; temporary psychiatric disability pension (full time or partial) [TDP(P)]; non-psychiatric (“other”) permanent disability pension (OPDP); non-psychiatric temporary disability pension (OTDP); sickness allowance (S); student (ST); unemployed (U); work (W); or death (D). If more than one state was identified at the same time, these were combined and labelled with the label of a joint state or of the dominating state. The dominating states were defined with the following rules: (a) death dominates all states, (b) permanent psychiatric disability pension (full time or partial) PDP(P) dominates all states except death, (c) temporary psychiatric disability pension (full time or partial) TDP(P) dominates all states except death and PDP(P), (d) non-psychiatric disability pension OPDP dominates all states except death, PDP(P) and TDP(P), (e) sickness allowance dominates unemployment and student, and (f) work and unemployed both dominate students. State distributions over time were computed and the individuals were clustered on the basis of their individual temporal sequences of states. Optimal matching was used as the clustering algorithm with constant distances (distance = 2) between the states, with the following exceptions: a missing state was considered to be closer to any other state (distance = 1) and pensions with the same basis were considered closer to each other (distance = 1), and mutually intermediately close (distance = 1.5). Individuals who had missing data at > 20% of the times were excluded from the clustering procedure, and thus, the states of 33,656 individuals were included in the clustering analysis. The data rules drive the hierarchical clustering algorithm away from deriving non-interpretable clusters. The choice of the number of clusters presented was based on the height of consecutive steps in the dendrogram as well as on interpretability: after seven clusters the qualitative meanings of the clusters started to overlap. We also verified that there were no clusters with atypical association in outcome analyses among the first 20 clusters derived from the associated dendrogram (data not shown).

Analysis of return to work (RTW) after psychiatric disability leave was based on the current state of working at points from 1–5 years from granting of the originally temporary pension. The probability of RTW was modelled with a generalized estimating equation framework with a logistic link. The unadjusted model investigated the probabilities of RTW for each individual assigned to a cluster on the basis of the temporal sequence of states prior to the pension. The probability of RTW was modelled as a quadratic function of time for each cluster. The adjusted model corrected for confounding factors: age group, gender, and diagnosis. These factors were included as main effects, as there were no interactions between them and time.

Survival analysis was conducted with the proportional hazards regression model. Cluster membership prior to pension was the primary explanatory factor in the model, and the analysis was adjusted for the same confounding factors as the RTW analyses. We did not observe deviations from the model assumptions.

The sequence analysis was conducted with the TraMinerR package [15], and the outcome analyses with gee and survival packages in R, version 3.5.1.

### Ethical issues

The study is based on register data collected for administrative, development, and evaluation purposes on a regular basis. No patients were contacted individually, and none will be recognizable from the data. The Ethical Committee of the National Institute of Welfare and Health gave its approval of the plan of the project.

## Results

### Background characteristics

From our comprehensive register databases, we found 42,170 persons who had been awarded either a temporary or permanent full or partial disability pension primarily due to a psychiatric disorder, starting as a new episode between 2010 and 2015. Overall, the mean age of the subjects was 42 years (SD 13.6; IQR 30 years; 54 years), and 45% of them were males. Regarding the major diagnostic categories, the most common psychiatric causes for retirement were affective disorders (F30–39): 63.9%, followed by psychotic disorders (F20–29): 17.2%.

### Labour market states prior to the psychiatric disability pension

**Fig. 1:**
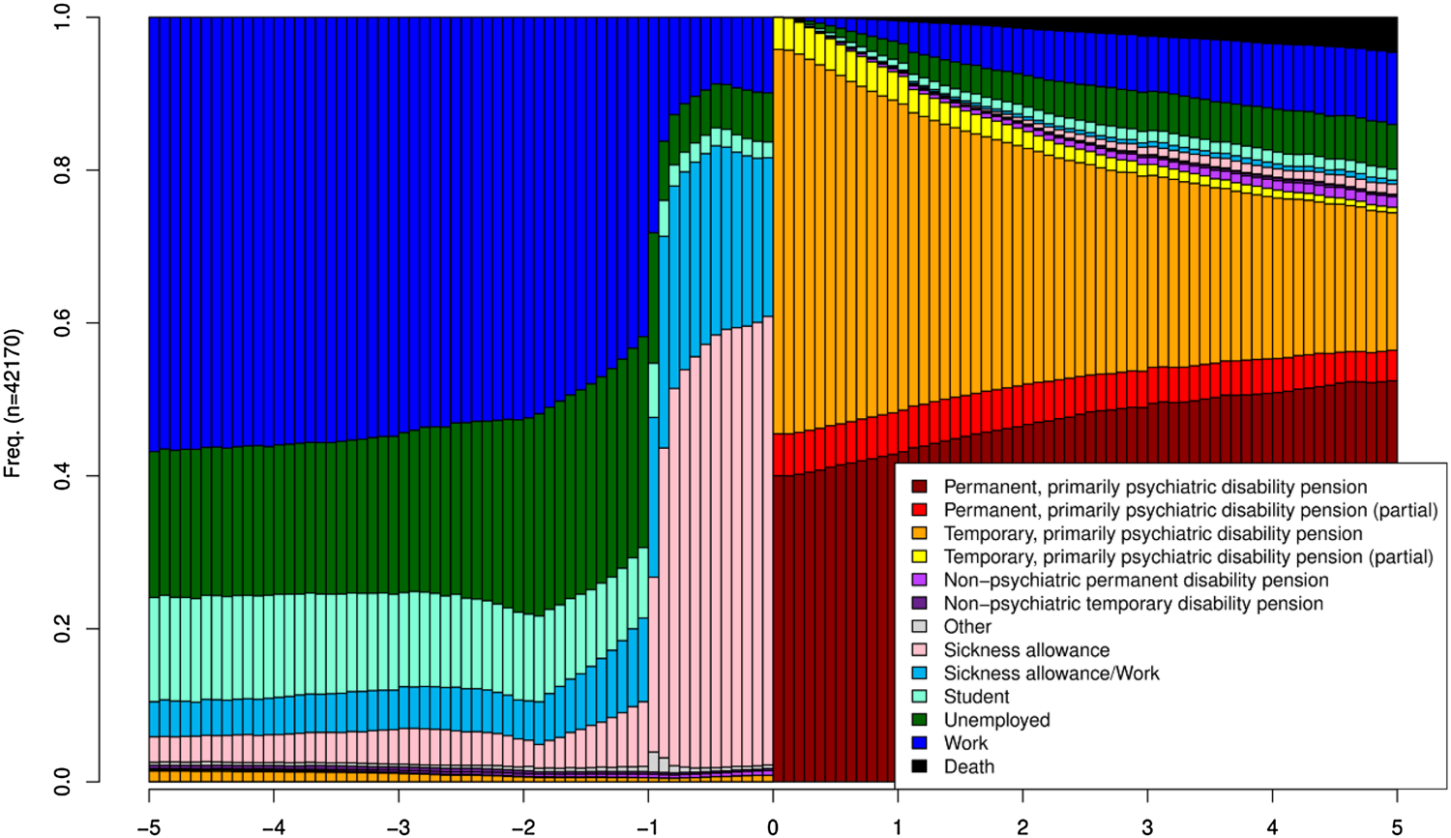
Labour market state, disability, and death among the study population. The time of the first psychiatric disability pension is shown in the middle (at 0 years of follow-up)

Labour market or disability (including death) states prior to and after the first awarded psychiatric disability pension are shown in Fig. 1. The distribution of labour market states was rather stable over time until 1 year prior to the first awarded psychiatric disability pension. Of those with known states, during that 4-year period, more than one half were employed, approximately one-fifth were unemployed, and one-tenth were students. A change point in the most frequent states was observed at 1 year before the disability pension, showing a rapid increase in the proportion of persons receiving sickness allowance. Approximately 80% of the individuals with known states were on sickness allowance during each month of the previous year. However, due to within-subject transitions between the states, only a very small proportion of subjects had their pension granted without any preceding sickness allowance period.

Immediately after the disability pension, temporary and permanent full psychiatric disability pensions represented more than 90% of the states. The remaining individuals had partial temporary or permanent pensions. Over time, there was a little change in the proportion of permanent partial pensions, but a modest increase in the proportion of permanent full pensions. At 5 years, approximately 20% were no longer pensioned. The most common other states were return to work, unemployment, or death, in descending order of frequency.

### Paths to disability pension

**Fig. 2:**
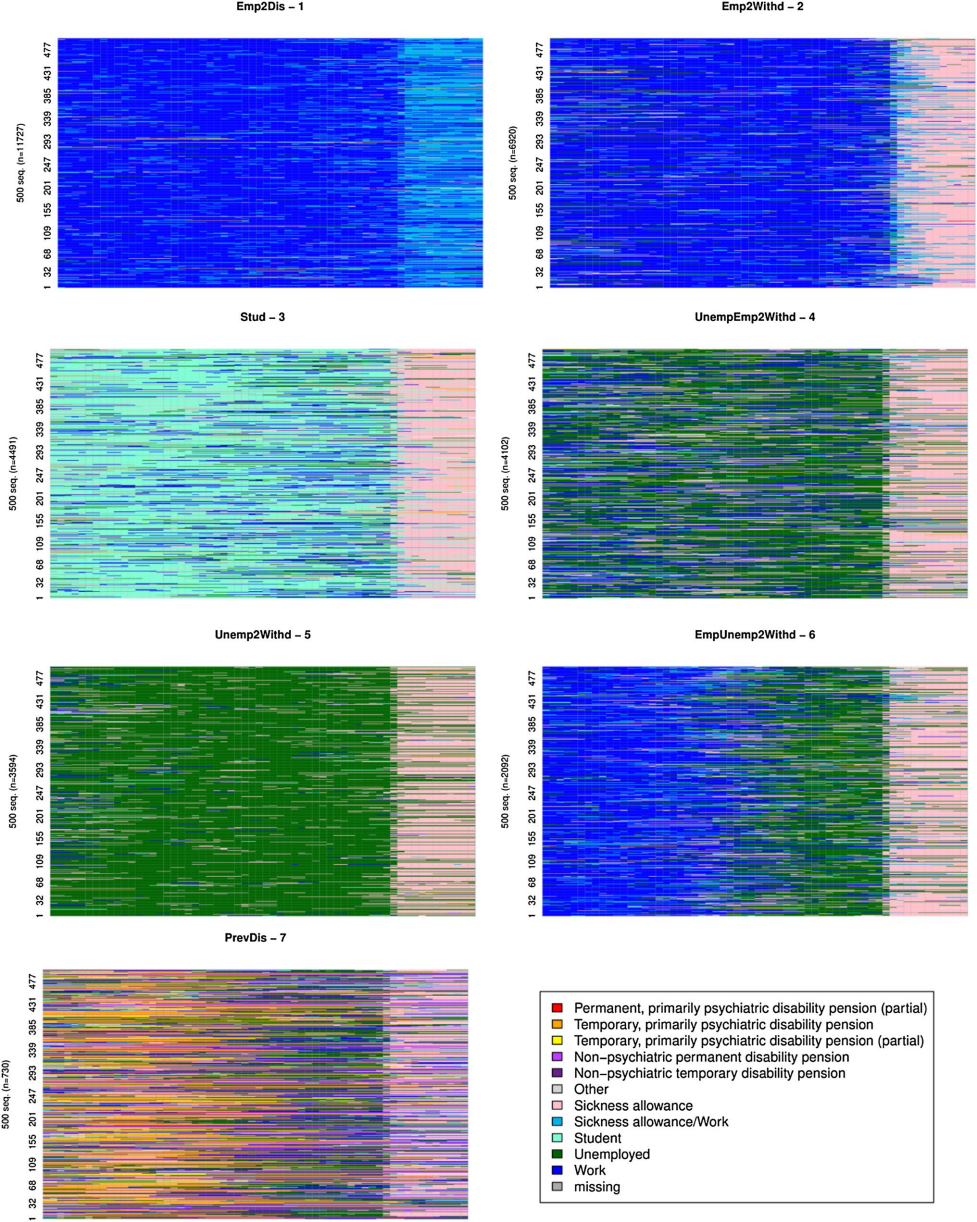
Seven distinctive paths to disability obtained by clustering of individual state sequences. The figure shows 500 sequences prior to retirement within each cluster

Altogether 33,656 subjects had at least 80% of state information available and were eligible for the clustering of their state sequences. In the chosen seven-cluster solution, the most common path to disability pension was being full-time employed up to the year prior to the first awarded disability pension, and then spending the required 300 days on sick leave away from work, during which period the employment relationship continued. The second cluster included predominantly subjects with employment ending before a period of sick leave. The third largest cluster consisted of students transferring to disability retirement through a sickness allowance spell. Another frequently occurring pattern was being unemployed, and shifting to an awarded disability pension either through temporary sick leave while unemployed, or directly (Fig. 2).

The most common state transitions in each cluster are reported in Table 1. 35% of the subjects were assigned to the largest cluster, among which the most common path was to move from employment via sick leave to a disability pension (Emp2Dis cluster). The second largest cluster, consisting of 21% of the subjects, was characterized by discontinuing employment before sick leave and disability pension (Emp2Withd). The third largest cluster consisted of students (13%). Qualitatively different clusters were characterized by long unemployment (Unemp2Withd, 11%) and the cluster with previous periods of non-psychiatric disability (PrevDis, 2%). Two other clusters could be described with alternating states of employment and unemployment before sick leave (UnempEmp2Withd, 12%), and from employment to unemployment followed by sick leave (Emp2Unemp2Withd, 6%) (Table 1).

**Table 1:** Characteristics of clusters identified in the analysis

Code	Emp2Dis	Emp2Withd	Student	UnempEmp2Withd	Unemp2Withd	EmpUnemp2Withd	PrevDis
Number of subjects (%)	11,727 (34.8)	6920 (20.6)	4491 (13.3)	4102 (12.2)	3594 (10.7)	2092 (6.2)	730 (2.2)
The most common sequence	Employment to disability	Employment to sick leave	Student	Mixed unemployment and employment to sick leave	Unemployment to sick leave	Employment to unemployment to sick leave	Previous non-psychiatric disability
Dg: F10–19 (%)	0.7	1.4	0.2	4.9	14.3	3.3	3
Dg: F20–29 (%)	6.7	14	30	18.7	19.2	16	18.5
Dg: F30–39 (%)	82.6	77.3	47.8	61.6	46.3	70.3	65.9
Mean age, years	49.8	44.6	24.3	44.9	47.3	46.4	43.8
Males (%)	31.4	48.2	44.7	47	59	47.3	39.9
Return to work force 5 years, %	16.3	13.8	10.8	3.5	1.2	4.4	7.8
Unemployed 5 years, %	3.1	6.2	9.8	6.8	4.3	6.2	5.8
Permanent disability 5 years, %	49.9	51.5	18.5	67.4	73.7	66.7	58.9

Notable differences between the clusters could be seen in the distribution of diagnoses: 83% of the largest (Emp2Dis) cluster members had a diagnosis of affective disorders (F30–39). Only 46% of the subjects in the Unemp2Withd (predominantly unemployment) cluster had a diagnosis of the same category. Psychotic diagnoses (F20–F29) were relatively more frequent in the student cluster than in other clusters. F10–F19 diagnoses as principal ones were rare overall, but the Unemp2Withd cluster showed a markedly higher proportion of this diagnostic category than other clusters (Table 1).

The proportion of women was highest in the Emp2Dis (predominantly employed) cluster (cluster 1) and the proportion of males was highest in the UnEmp2Withd cluster. On average, the student cluster had by far the youngest members, and the Emp2Dis cluster the oldest (Table 1).

### Permanent disability, return to work (RTW), and unemployment

After the retirement, an increasing proportion of subjects became permanently disabled (Fig. 1). Overall, whereas around 40% of subjects were on full-time permanent disability pension from the beginning of the first pension award, half of the subjects were in this state 5 years after the retirement. The proportion was by far the lowest in the student cluster that was also the youngest cluster, on average (Table 1). Clusters characterized by unemployment periods before the retirement had the highest rates of permanent full-time disability at 5 years.

Return to work (RTW) was not common in the study population overall, as only 10.5% were in the state after the 5-year follow-up. In addition, we found notable differences between the clusters in the proportion of subjects who returned to work after a disability pension (Fig. 3 in Supplement Material). Distinct paths to disability depicted by the seven clusters were strong predictors of subsequent RTW: the Emp2Dis cluster showed the highest rate of RTW, which was significantly different from all other clusters (Table 2). Unemp2Withd and UnempEmp2Withd clusters had the poorest rates of return to work, with 5-year adjusted odds ratios of 0.09 (95% CI 0.05, 0.15) and 0.18 (0.13, 0.26), respectively. Student and PrevDis clusters, which represent special sub-populations, also had substantially lower RTW rates than were observed in the Emp2Dis cluster. These differences could not be explained by the differences in gender, diagnosis, or age distribution in the clusters (Table 2). In addition, the rate of return to work varied remarkably between different sub-populations: for example, in the Emp2Dis cluster, 32% of females (25% of males) aged 30–35 with an F30–F39 diagnosis returned to work, but only 12% (8.5%) of those aged 50–55 years did so. Similarly, and on the other extreme, members of the Unemp2Withd cluster with an F10–19 diagnosis had < 1% rates of RTW for both females and males for the same age groups.

**Table 2:** Results of the return to work analysis

Cluster	Return to work						Odds ratio (95% CI) at 5 years^a^	
	At 1 year		At 3 years		At 5 years		Unadjusted	Adjusted^b^
	*n/N*	(%)	*n/N*	(%)	*n/N*	(%)		
Emp2Dis	540/10,278	5.3	846/6205	13.6	369/2262	16.3	Ref.	Ref.
Emp2Withd	232/6102	3.8	408/3967	10.3	224/1619	13.8	0.90 (0.76, 1.10)	0.80 (0.67, 0.95)
Student	78/3792	2.1	182/2367	7.7	98/909	10.8	0.68 (0.55, 0.86)	0.36 (0.29, 0.46)
UnempEmp2Withd	31/3663	0.8	66/2465	2.7	35/1014	3.5	0.20 (0.14, 0.28)	0.18 (0.13, 0.26)
Unemp2Withd	13/3166	0.4	15/2078	0.7	10/857	1.2	0.07 (0.04, 0.13)	0.09 (0.05, 0.15)
Emp2Unemp2Withd	16/1873	0.9	42/1286	3.3	19/435	4.4	0.22 (0.13, 0.35)	0.20 (0.12, 0.34)
PrevDis	11/694	1.6	20/542	3.7	20/258	7.6	0.49 (0.31, 0.76)	0.40 (0.25, 0.62)

^a^Estimates derived from the GEE model

^b^Adjusted for age group, gender, and diagnosis

*n* number of employed subjects, *N* number of subjects in follow-up

Five-year unemployment rates varied between 3.1 and 9.8% between clusters; i.e., the rates did not differ from one cluster to another as much as did the permanent disability rates.

### Mortality

Altogether 1085 deaths occurred among the subjects during the follow-up and 768 among those who were eligible for clustering of the paths to disability. Between clusters, mortality varied, being highest among the Unemp2Withd cluster (characterized by long-term unemployment), and lowest among the Emp2Dis and Student clusters. The 5-year mortality rates by paths to disability were seen to show a clear deviation of the UnEmp2Withd cluster from the others, in a separate survival graph (Supplementary Fig. 4).

Age, diagnosis, and gender were controlled for in a Cox regression model estimating the relative risk of death between the pre-retirement clusters of labour market histories. The unadjusted results show marked differences in mortality, dependent on the path to disability. Due to unbalance of confounding factors across clusters, age, gender, and diagnosis explain a substantial proportion of these differences, but not all of them (Table 3). Altogether, the retired males had more than twofold mortality compared to females [hazard ratio (HR) 2.7 (95% CI 2.3, 3.1)]. Even after adjustment for the confounding factors, the Unemp2With cluster stood out with a 1.7-fold risk of death. Two other clusters with unemployment periods also showed increased mortality of almost the same magnitude.

**Table 3:** Results of the survival analysis

Cluster	Number of deaths	5-year survival rate^a^	Hazard ratio (95% CI)	
			Unadjusted	Adjusted
Emp2Dis	181	97.1%	1 (reference)	1 (reference)
Emp2Withd	165	95.9%	1.45 (1.17, 1.79)	1.37 (1.11, 1.70)
Student	46	98.2%	0.66 (0.48, 0.91)	1.13 (0.71, 1.80)
UnempEmp2Withd	129	94.7%	1.87 (1.49, 2.34)	1.52 (1.20, 1.92)
Unemp2Withd	192	91.0%	3.34 (2.73, 4.09)	1.73 (1.38, 2.17)
EmpUnemp2Withd	64	95.1%	1.83 (1.38, 2.44)	1.58 (1.18, 2.10)
PrevDis	21	96.4%	1.42 (0.91, 2.23)	1.45 (0.92, 2.28)

Adjustments were made for age group, gender, and diagnosis

^a^Kaplan–Meier estimate

## Discussion

In our comprehensive data set covering psychiatric disability retirement processes in an established Northern European social insurance system, we found varying, distinguishable paths from initial disability to lost work ability and to possible further return to work or death. A remarkably small proportion of persons who were granted temporary retirement for psychiatric reasons eventually returned to work within a 5-year follow-up. We observed excess mortality among the males in the population. Preceding unemployment was a major risk factor for either permanent retirement or death: those with long-term unemployment had minimal chances of returning to work and demonstrated increased mortality within the follow-up period.

In a cluster analysis of the labour market states, the most common pattern before premature retirement for psychiatric reasons was being employed until being granted a temporary or permanent disability pension. In this cluster of subjects, mortality in the follow-up was markedly lower than in other groups. In contrast, preceding long-term unemployment carried an increased risk of death following retirement. Although, among the unemployed, their preceding physical health status may have increased the probability of psychiatric disability and retirement, their elevated mortality indicates a possible lack of health and social care, including suicide prevention [16, 17]. On the other hand, in case of cardiovascular reasons, the role of long-term psychotropic medication and lifestyle factors should be recognized, too [18].

The cluster of predominantly employed subjects—characterized by a relative abundance of females, affective disorders, and higher age—had better outcomes in terms of regaining their occupational capacity. Although not evidenced by the present data, their paths of functional loss may have been shorter and more reactive to changes or setbacks in occupational or personal life, than the cluster of the predominantly unemployed subjects, often disturbed by psychotic or substance use disorders. The better occupational outcomes (RTW) of this cluster are in accordance with earlier research, which has pointed out the role of preceding employment as a predictor for a positive outcome [9]. We are now suggesting that in this case, several characteristics associate with and perhaps contribute to more frequent RTW simultaneously.

While those losing their active work ability due to mental disorders may be prone to return to work after active rehabilitation, particular attention should be paid to those with long-term unemployment. In the case of prolonged unemployment, it is worth considering whether disability retirement and possible preceding rehabilitating efforts should have taken place much earlier. Already during their unemployment, these subjects should rather have been on disability pension than primarily unemployed. From another perspective, people with severe mental health disorders are likely to drop out of the active labour market or never enter it, and they are at risk of becoming as long-term unemployed without further notice. One Finnish register study already earlier reported the impact of mental disorders on increased mortality and possible poorer quality of patients’ health care in general [19].

Non-disease-related factors affecting the incidence of disability retirement include municipal socioeconomic-, demographic-, work-related, and labour force characteristics [20–26]. Long-term treatment itself may have harmful effects, too [17]. The process of awarding pensions is centralized, but known regional differences suggest that varying rehabilitating activity and other service-related factors may affect the rate of return to work (RTW) [9, 21], and should be analysed further in the future. A particularly interesting group is those subjects partially returning to work for different reasons. They are not seen in this setting, due to the dominance of other labour market states, but will be studied in more detail separately.

The statistical sequence analysis approach allowed us to characterize the main paths in the study population according to their employment or other activity status [27]. The mental disorder diagnosis and other confounding factors were taken into account in the multivariate analysis of the probabilities of (1) return to work and (2) survival. As expected, adding the effect of a mental disorder attenuated the associations between the cluster and the outcome, but they remained both significant and meaningful. This points towards the separate effects of mental disorder per se, but it is particularly noteworthy that the labour market history has an independent predictive role.

### Service system implications

Our findings illustrate the diversity of the population losing their occupational capacity due to mental disorders. Mental disorders impact personal functioning in a variety of ways and settings. Psychotic disorders are known to affect individuals already at a young age, leading most probably to a poor long-term attachment to the work force, whereas affective disorders usually have their impact later in working life. When controlled for sociodemographic and diagnostic factors, our results highlight that it is possible to identify characteristics that may indicate a potential recovery of work ability. Individuals with an active working career who discontinue because of an affective illness (often depression) could more probably be actively and successfully treated and rehabilitated towards a part-time or even full-time return to work. On the other hand, it seems that our service system has difficulties in rehabilitating persons suffering from substance use disorders with related unemployment.

Subjects with prolonged unemployment and subsequent disability appear to be more difficult to return to the work force by rehabilitating than those with preceding working periods. Their excess mortality should be taken into account in terms of health care efforts. In fact, a significant proportion of these subjects are most probably occupationally disabled already long before the actual retirement. Our system probably fails to recognize this, leading to labelling some of the truly disabled individuals as unemployed.

Conversely, one indication for successful rehabilitation is an active working life before retirement, particularly among females. This potential should be recognized early enough, before assigning the first temporary granted pensions. Vocational rehabilitation might be particularly justified in the case of these subjects, thus rendering earlier positive outcomes possible. The positive role of work for the mental health of individuals is being increasingly recognized in national-level strategic planning [28].

### Research implications

Our approach to the analysis of comprehensive register data allowed us to combine the longitudinal employment and disability data with the diagnostic and sociodemographic data, to characterize the people at elevated risk for permanent disability or death. By integrating the graphical analysis with inferential analysis, we obtained a realistic and visual overview of the differing retirement patterns, and their association with outcomes that have public health and well-being as well as economical relevance. Such patterns could, perhaps, even be automatically recognized from our electronic systems in the future, which could act as a signalling system for more intensive interventions.

## Conclusions

Retirement is a complex process involving identifiable clinical and contextual associating factors. Labour market history patterns are associated with varying prognoses after psychiatric retirement. Prolonged unemployment appears as a predictor of relatively poor prognoses, whereas preceding steady employment indicates the opposite.

## Electronic supplementary material

Below is the link to the electronic supplementary material.

Supplementary material 1 (DOCX 71 kb)
