# Supplementary material for: The importance of clinical and labour market histories in psychiatric disability retirement: analysis of the comprehensive Finnish national-level RETIRE data
Source: Soc Psychiatry Psychiatr Epidemiol. 2019 Dec 5;55(8):1011–20. doi: 10.1007/s00127-019-01815-6 (PMC7394924; doi:10.1007/s00127-019-01815-6)
Supplement: Supplementary file 1 — Supplementary material 1 (DOCX 71 kb) [file 127_2019_1815_MOESM1_ESM.docx]

**The importance of clinical and labour market histories in psychiatric disability retirement - analysis of the comprehensive Finnish national level RETIRE data**

Pirkola S, Nevalainen J, Laaksonen M, Fröjd S, Nurmela K, Näppilä T, Tuulio-Henriksson A, Autio R, Blomgren J

*Supplementary material*

*Figure 3: proportions of subject at work (return to work) by paths to disability and time from awarding the disability pension.*

*Figure 4 Survival rates by paths to disability.*
